# Checklist of pioneer benthic taxa found on Autonomous Reef Monitoring Structures (ARMS) in Terra Nova Bay (Ross Sea, Antarctica)

**DOI:** 10.3897/BDJ.13.e148863

**Published:** 2025-04-08

**Authors:** Valentina Cometti, Matteo Cecchetto, Alice Guzzi, Marco Grillo, Nicholas Francesco Noli, Simonetta Corsolini, Stefano Schiaparelli

**Affiliations:** 1 Department of Physical, Earth and Environmental Sciences, University of Siena, Siena, Italy Department of Physical, Earth and Environmental Sciences, University of Siena Siena Italy; 2 Italian National Antarctic Museum (MNA, section of Genoa), Genoa, Italy Italian National Antarctic Museum (MNA, section of Genoa) Genoa Italy; 3 Department of Earth, Environmental and Life Sciences (DISTAV), University of Genoa, Genoa, Italy Department of Earth, Environmental and Life Sciences (DISTAV), University of Genoa Genoa Italy; 4 National Biodiversity Future Center (NBFC), Palermo, Italy National Biodiversity Future Center (NBFC) Palermo Italy; 5 Institute of Polar Sciences, Italian National Research Council (ISP-CNR), Bologna, Italy Institute of Polar Sciences, Italian National Research Council (ISP-CNR) Bologna Italy

**Keywords:** distributional occurrences, check-list, time series, ARMS, Southern Ocean, Ross Sea, Terra Nova Bay, Italian National Antarctic Museum (MNA), biodiversity

## Abstract

**Background:**

Benthic communities studies in the Southern Ocean highlight their potential for assessing climate and anthropogenic impacts. However, the lack of standardised methods limits result reliability and interpretation. This dataset presents the first checklist focus on the Antarctic pioneer benthic communities collected using a standardised approach such as Autonomous Reef Monitoring Structures (ARMS) located at 25 m depth in the surroundings of the Italian research station "Mario Zucchelli" (MZS) in the Terra Nova Bay (TNB) area of the Ross Sea, Antarctica. The data encompass ARMS time series corresponding to deployments of 1, 2, 3 and 5 years, from which 277 occurrence data corresponding to 12 phyla, 43 families, 49 genera and 39 species were obtained. All retrieved specimens are curated by the Italian National Antarctic Museum (MNA, section of Genoa). This dataset is a contribution to the Antarctic Biodiversity Portal, the thematic Antarctic node for both the Ocean Biogeographic Information System (AntOBIS) and the Global Biodiversity Information Facility Antarctic Biodiversity Information Facility (ANTABIF). The dataset was uploaded and integrated with the SCAR-AntOBIS database under the licence CC-BY 4.0. Please follow the guidelines from the SCAR Data Policy (ISSN 1998-0337) when using the data. If you have any questions regarding this dataset, please contact us via the contact information provided in the metadata or via data-biodiversity-aq@naturalsciences.be. Issues with the dataset can be reported at the biodiversity-aq GitHub project.

**New information:**

We describe the biodiversity of the Antarctic pioneer benthic communities of TNB sampled using the ARMS installed at the Italian research station "Mario Zucchelli". ARMS is a standardised, reproducible and comparable method for quantifying biodiversity. This dataset provides essential baseline data on the occurrence and abundance of pioneer benthic communities in this study area, representing an important contribution for understanding the dynamics of benthic pioneer communities in an area where these structures have never been deployed and, in general, for an exposure time that largely exceed the standard one, which is usually of one year only.

The 277 occurrences reported here have been classified at the lowest possible taxonomic level and comprise 39 recognised species, 49 genera and 43 families. Approximately 98% of the samples are stored in 96% ethanol, while the others at -20°C, representing a potential resource for future genetic studies. To date, the entire ARMS collection has not been DNA barcoded, although preliminary metabarcoding analyses have already been published in Cecchetto et al. (2024). Outcomes of the barcoding activity will be the target of another future publication (Cometti et al., in prep). The publication of this data paper was funded by the Belgian Science Policy Office (BELSPO, contract n°FR/36/AN1/AntaBIS) in the framework of EU-Lifewatch as a contribution to the SCAR Antarctic Biodiversity Portal (bio diversity.aq).

## Introduction

The Southern Ocean has unique environmental conditions compared to other areas of the Planet and is characterised by a high degree of endemism (De Broyer et al. 2014), high levels of cryptic diversity at the species level (Wilson et al. 2009, Maroni and Wilson 2022, Maroni et al. 2022) and a Palaeozoic functional grade of organisation (Aronson and Blake 2001). To date, the available data on the development and dynamics of benthic communities have shown interesting potential for understanding the impacts of anthropogenic and climate change on them, showing changes at both community and species levels (Barnes et al. 2021).

Antarctic hard substrates fouling communities of Terra Nova Bay (TNB) were initially studied during the expeditions 1987-88, 1989-90 and 1993-94, conducted by the Italian National Antarctic Research Program (PNRA) (Cattaneo-Vietti et al. 2000). During the same period, other areas of the continent, such as McMurdo Sound (Ross Sea), were investigated (e.g. Dayton (1989)).

Since these initial studies, there have been no new attempts to study pioneer benthic communities until recently, when new investigations into the colonisation of artificial substrates (e.g. Bowden et al. (2006), Dayton et al. (2016), Caruso (2019), Barnes et al. (2021)) and natural substrates (e.g. Barnes and Souster (2011), Fillinger et al. (2013), Barnes et al. (2014), Krzeminska and Kuklinski (2018)) were undertaken. To date, the data show a very critical and slow growth rate (Peck 2018) and reveal the presence of various taxa including Porifera, Cnidaria, Annelida, Mollusca, Bryozoa, Brachiopoda, Chordata, Algae (Arntz and Gallardo 1994, Bowden et al. 2006) and bacterial communities (Caruso et al. (2023), Papale et al. (2024)).

However, these studies have never employed reproducible and standardised techniques, limiting both the reliability of the results and the broader understanding of observed changes. A standardised approach is essential to ensure the replicability of analyses, a key feature of biological monitoring at regional and global scales.

This feature is provided by the Autonomous Reef Monitoring Structures (ARMS), designed by Leray and Knowlton (2015). The ARMS units, part of the Global ARMS Programme, consists of ten 22.5 × 22.5-cm PVC plates stacked on top of each other, simulating a complex 3D environment. Furthermore, these structures present alternating layers of crevices open and closed to the flow of the current. ARMS provide a quantitative, reproducible, standardised and cost-effective method that enables reliable comparisons across studies (Leray and Knowlton 2015, David et al. 2019, Pearman et al. 2020).

The simplified design of these structures provides an easily quantifiable sampling methodology that, combined with the study of fouling organisms with High Throughput Sequencing (HTS), became a standard in monitoring activities at sea (Valentini et al. 2016) allowing communities to be inspected in different time intervals and environmental conditions (Cecchetto et al. 2024). ARMS are a powerful tool to obtain information on the likely resilience of the benthic fauna in relation to possible changes and to significantly improve the accuracy and feasibility of monitoring efforts (Fonseca et al. 2017), especially in the Ross Sea, where they have never been adopted.

This dataset presents the first checklist of pioneer benthic organisms in TNB, which have never been studied using these structures and over such a long monitoring time. Previous MNA contributions focused on Mollusca, Tanaidacea, Fungi, Ophiuroidea, Porifera, Bryozoa, Rotifera, Asteroidea, Copepoda and Isopoda (Ghiglione et al. 2013, Piazza et al. 2014, Selbmann et al. 2015, Cecchetto et al. 2017, Ghiglione et al. 2018, Garlasché et al. 2019, Cecchetto et al. 2019, Bonello et al. 2020, Guzzi et al. 2022, Grillo et al. 2024, Noli et al. 2024).

The special issue that included this publication contains additional articles that centre on specific marine animals, such as Holothurians (Guzzi et al., in prep), Amphipods (Cecchetto et al., in prep) and fish (La Mesa et al., in prep). This dataset also represents another Italian contribution to the CCAMLR CONSERVATION MEASURE 91-05 (2016) for the Ross Sea region Marine Protected Area, specifically addressing Annex 91-05/C (“long-term monitoring of benthic ecosystem functions”).

## Project description

### Title

Checklist of pioneer benthic taxa found on Autonomous Reef Monitoring Structures (ARMS) in Terra Nova Bay (Ross Sea, Antarctica)

### Personnel

Valentina Cometti, Matteo Cecchetto, Alice Guzzi, Marco Grillo, Nicholas Francesco Noli, Simonetta Corsolini, Stefano Schiaparelli

### Study area description

**Figure 1.**
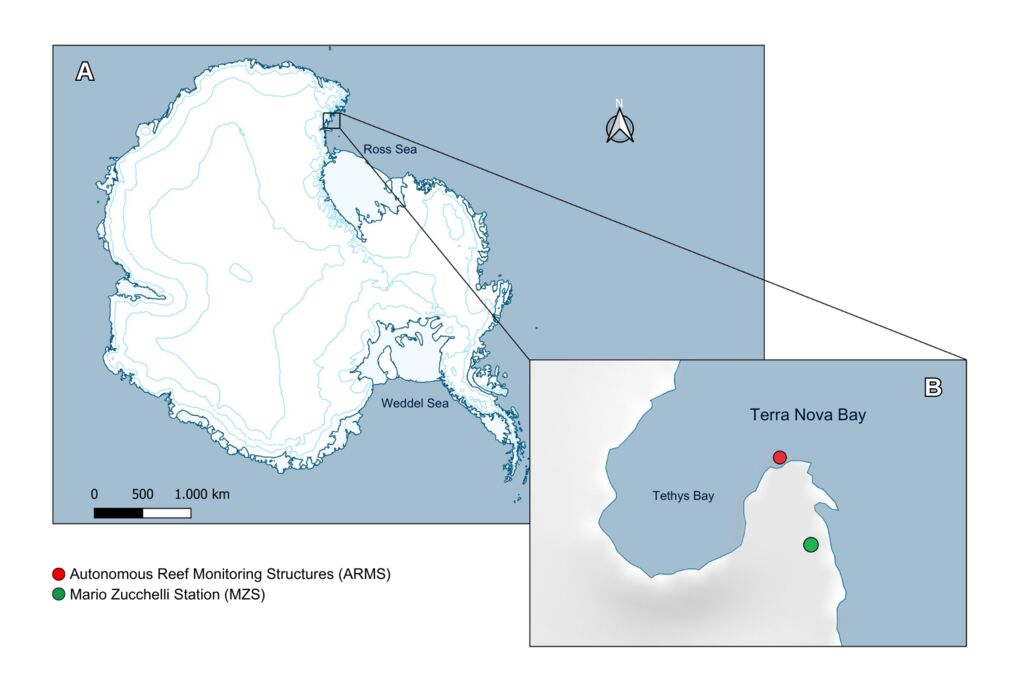
Overview of Antarctica (**A**) and in zoomed view of Tethys Bay (within Terra Nova Bay, Ross Sea) (**B**).

The occurrence data of the pioneer benthic communities studied in this data paper derives from the XXXII, XXXIII, XXXIV, XXXVII and XXXVIII Expeditions of the Italian National Antarctic Program (PNRA). Samples were collected from ARMS located at a depth of 25 m at the ‘Zecca’ site in Tethys Bay (-74.690°, 164.103°), approximately 500 mfrom the Mario Zucchelli Station (Fig. 1), in the TNB area. The sampling period spanned from 1 December 2015 to 8 November 2022. The seabed surrounding the ARMS consists of heterogeneous, unsorted sediments including sand, gravel and small cobbles mainly colonised by Corallinales. The area is characterised by a high abundance of *Sterechinusneumayeri* (Meissner, 1900) and *Odontastervalidus* Koehler, 1906 (Piazza et al. 2019, Piazza et al. 2020), which were frequently observed at the same site during the retrieval of the structures.

### Funding

Data originated in the framework of six different PNRA (Italian National Antarctic Program) expeditions carried out from 2015 to 2022. The deployment, recovery and analyses of the ARMS deployed in TNB were funded by the Italian National Antarctic Program (PNRA) projects:

- “TNB-CODE - Terra Nova Bay barCODing and mEtabarcoding of Antarctic organisms from marine and limno-terrestrial environments” (Project code 2016/AZ1.17; PI Prof. Schiaparelli S.).
- “RosS-MODe – Ross Sea biodiversity Monitoring through barcoding, metabarcODing and e-DNA” (Project code PNRA18_00078, PI Prof. Ficetola F.).

The publication of this data paper was funded by the Belgian Science Policy Office (BELSPO, contract n°FR/36/AN1/AntaBIS) in the Framework of EU-Lifewatch as a contribution to the SCAR Antarctic biodiversity portal.

## Sampling methods

### Sampling description

**Figure 2.**
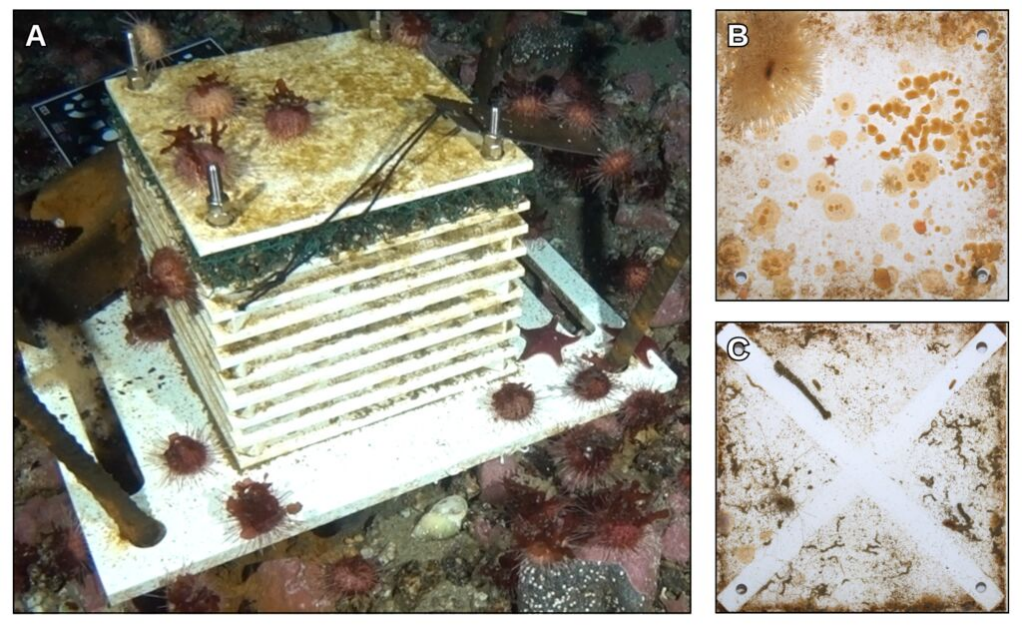
Photo extracted from underwater video showing an ARMS deployed in the sampling area (**A**). The structure was recovered during the XXXVII PNRA expedition in 2021 (after 5 years of colonisation). After recovery, photographs were taken of the PVC panels. In detail, we see panel number 7 facing downwards (**B**) and panel number 5 facing upwards (**C**).

Samples were collected using ARMS (Fig. 2). Each ARMS consists of 10 PVC plates (22.5 x 22.5 x 0.5 cm) stacked one top of each other and separated by 1 cm nylon spacers at the corners of each plate, into which four stainless steel bolts are threaded, holding the entire structure together. This is then fixed on top of a large 45 x 35 cm PVC base plate, which allows the whole structure to be anchored to the seafloor. ARMS have been recovered thanks to the help of PNRA SCUBA divers, which covered each retrieved structure with a rigid plastic crate perforated on each side and internally lined with a 100 μm nylon net, in order to avoid the escape of vagile benthic organisms. Other details available at Global ARMS Program site.

ARMS were deployed at sea for varying time ranges of 1, 2, 3 and 5 years. These six structures were recovered in different years: 2 in 2016 (1 year later), 2 in 2017 (2 years later), 2 in 2018 (3 years later). Additionally, one pair of ARMS was installed in 2016 and recovered in 2021 (5 years later) and another additional pair of ARMS was installed in 2017 and recovered in 2022 (5 years later).

### Quality control

All records were visually checked, identified at the lowest possible taxonomic level, validated and assigned an MNA voucher. Throughout all phases, quality control and data cleaning ensured high-quality data and reliable identifications. Throughout sorting, classification and storage at the MNA, quality control and data cleaning ensured high-quality data and reliable identifications. Coordinates were converted into decimal latitude and decimal longitude and plotted to verify the geographical location and locality. All scientific names were inspected for typos and were updated using the “WoRMS Taxon match” tool of the “World Register of Marine Species” (WoRMS) and AphiaID was assigned to each taxon as scientificNameID. The event dates and times were converted into ISO 8601 and verified with the field reports.

### Step description

**Figure 3.**
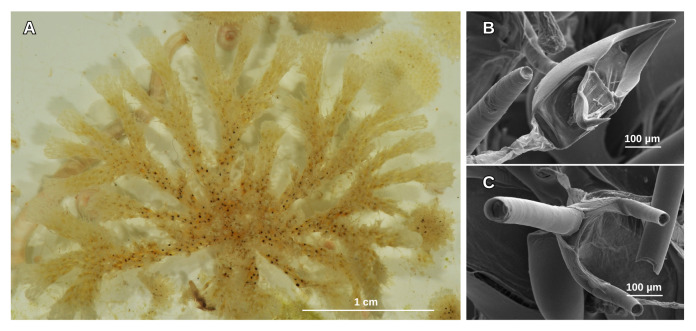
Sample of *Camptoplitestricornis* (Waters, 1904) (MNA-13822 voucher number) on PVC plate (Fig. 3A) identified using scanning electron microscopy (SEM). In detail, avicularia (Fig. 2B) and autozooid with spines (Fig. 2C). The sample is part of the material collected during the XXXIV PNRA Expedition (2018/2019).

The structures deployed in 2015 and recovered in 2016 (1 year), 2017 (2 years) and 2018 (3 years) were stored entirely at -20°C to be transported to Italy, where they were disassembled and analysed. After retrieval, each individual plate was appropriately photographed, from both sides, scraped and homogenised to form sub-samples that were subsequently stored in ethanol at -20°C. However, during the recovery of the first pair of structures (2016) and one of the second (2017), these crates malfunctioned and, thus, no quantitative analyses on the vagile component of the community inhabiting the ARMS could be performed. For the fifth-year structures, one pair deployed in 2016 and recovered in 2021 and the other pair deployed in 2017 and recovered in 2022, processing took place directly in Antarctica. The samples were sorted, acquired by the Italian National Antarctic Museum (MNA, Section Genoa) and directly stored in ethanol (96%) or at -20°C to be identified later. Most of the records were identified by one researcher, using original descriptions and taxonomic keys and the online WoRMS portal to confirm the acceptance of species names. The identification was often supported by scanning electron microscopy (SEM), combining stack images of the analysed specimens, particularly for the bryozoan specimens (Fig. 3). High-resolution SEM images were taken, focusing on diagnostic traits such as details of the primary orifice, ovicell morphology and other morphological characteristics. The light and contrast parameters were carefully adjusted to enhance specimen features, ensuring clarity for accurate identification. For bryozoan, a small portion of the colony of less than 1 cm was taken and treated with sodium hypochlorite (NaClO) for 10 minutes and washed with ethanol (EtOH) at different concentrations 70%, 90% and 100%. When identification was inconclusive, only genus or family names were assigned. The samples were deposited in the biological collection of the MNA. All data were uploaded to the GBIF portal.

## Geographic coverage

### Description

Samples were collected at one location, nominally "Zecca", from the Tethys Bay area. The sampling site is approximately 500 m distant from the MZS in TNB (Ross Sea, Antarctica) and at 25 m of depth (Fig. 1).

Coordinates of the deployment site: -74.690 Latitude, 164.104 Longitude

## Taxonomic coverage

### Description

**Figure 4.**
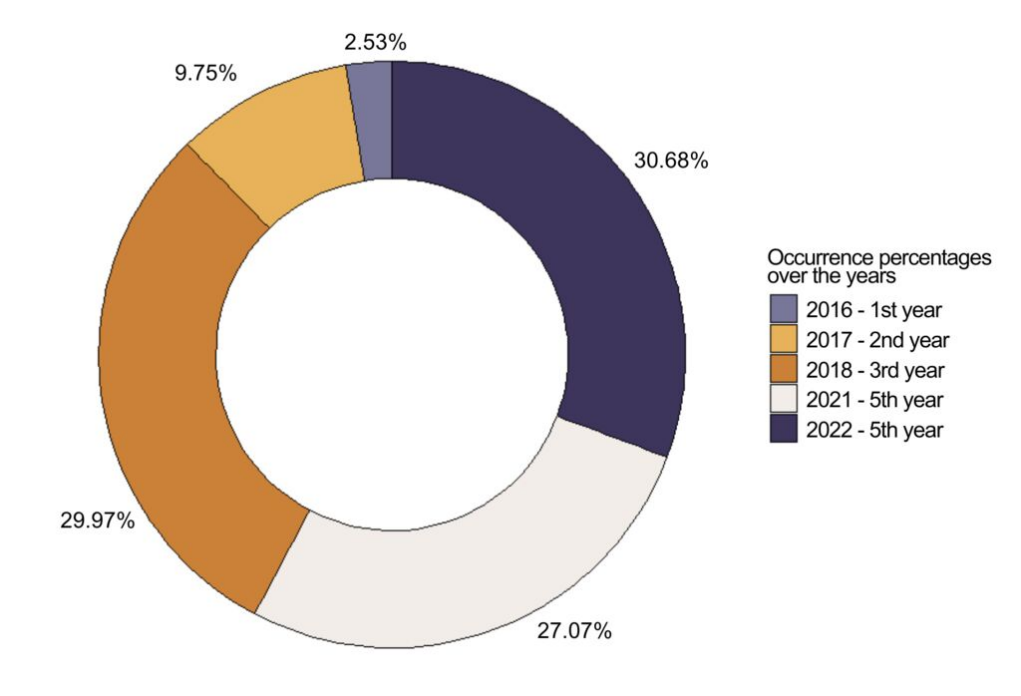
Occurrence percentages over the years from 2016 to 2022.

This dataset focuses on pioneer benthic taxa collected using ARMS. A total of 277 occurrences were recorded, with the largest proportions in 2022 (30.68%) and 2018 (29.97%), followed by 2021 (27.07%) and 2017 (9.75%). The smallest percentage was in 2016, accounting for only 2.53% of the total occurrences (Fig. 4).

**Figure 5.**
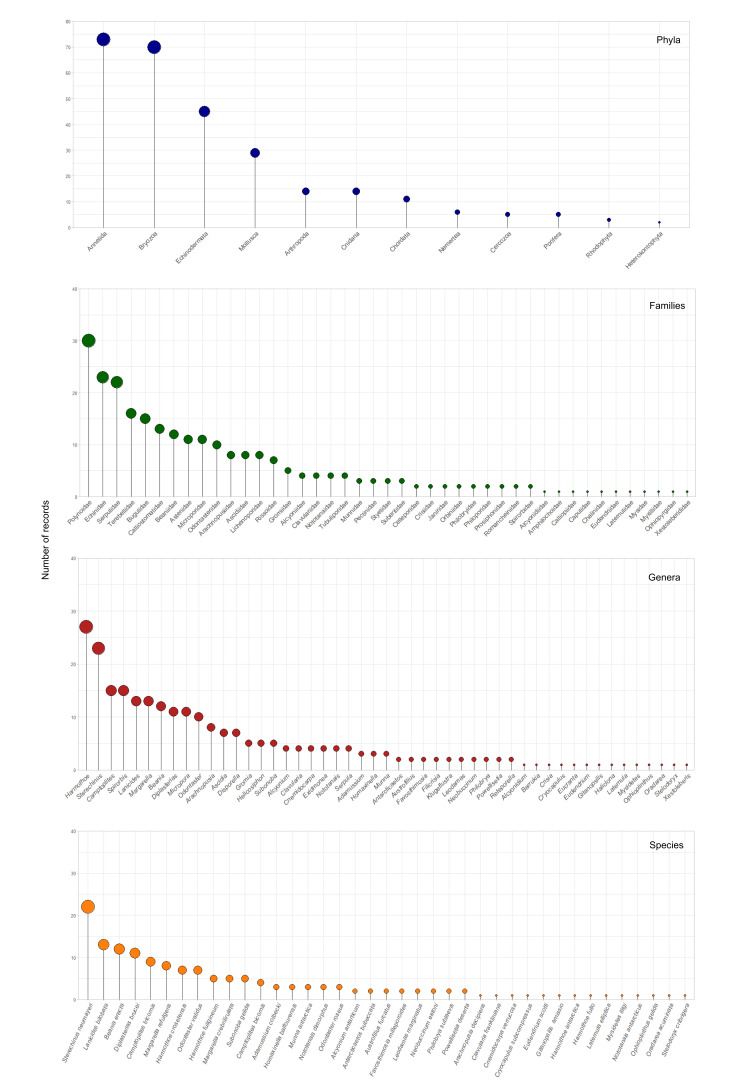
Number of records identified at the phylum, family, genus and species level, for each specific taxa.

After analysing the complete dataset of 277 occurrences, 43 families, 49 genera and 39 species were identified, along with 31 morphotypes. However, some taxa could not be identified to the species level and this uncertainty was indicated by 'sp.' or 'cf.' identification qualifiers in the dataset (Fig. 5). Futhermore, six specimens from the order Amphipoda were excluded from this dataset, as they are already listed in the checklist by Cecchetto et al. (in prep).

Of the 277 occurrences, 154 are sessile and 123 are vagile species.

Annelida is the most abundant phylum (73 occurrences), followed by Bryozoa (70 occurrences). While Echinodermata (45 occurrences) and Mollusca (29 occurrences) are also notable, they have fewer records compared to the top two phyla. Other phyla, such as Chordata and Porifera, have significantly fewer records (respectively 11 and 5), with the number of records gradually decreasing towards the least represented phyla.

The most representative families in the dataset are Polynoidae (30 occurrences), Echinidae (23 occurrences) and Serpulidae (22 occurrences). Amongst these, the most abundant genera were *Harmothoe* Kinberg, 1856 (27 occurrences) (POLYCHAETA, Polynoidae) and *Sterechinus* Koehler, 1901 (23 occurrences) (ECHINOIDEA, Echinidae). The species with the highest number of occurrences was *Sterechinusneumayeri* (Meissner, 1900), recorded 22 times.

After analysing the life stages of the specimens, a total of 277 occurrences were recorded, comprising 272 adults, three juveniles (corresponding to *Lanicidesbilobata* (Grube, 1877) and two speciments of *Adamussiumcolbecki* (E.A. Smith, 1902)) and two egg masses (*Neobuccinumeatoni* (E.A. Smith, 1875)).

Species with the symbol (*) in the following table indicate that they are new records for the TNB area. All the records recorded in this dataset are based on physical museum vouchers (hereafter “MNA collection records”) curated by the Genoa section of the MNA.

### Taxa included

**Table:** 

Rank	Scientific Name	
kingdom	Animalia	
kingdom	Chromista	
kingdom	Plantae	
phylum	Annelida	
phylum	Arthropoda	
phylum	Bryozoa	
phylum	Cercozoa	
phylum	Chordata	
phylum	Cnidaria	
phylum	Echinodermata	
phylum	Heterokontophyta	
phylum	Mollusca	
phylum	Nemertea	
phylum	Porifera	
phylum	Rhodophyta	
class	Ascidiacea	
class	Asteroidea	
class	Bacillariophyceae	
class	Bivalvia	
class	Demospongiae	
class	Echinoidea	
class	Gastropoda	
class	Gromiidea	
class	Gymnolaemata	
class	Hexacorallia	
class	Hydrozoa	
class	Malacostraca	
class	Octocorallia	
class	Ophiuroidea	
class	Ostracoda	
class	Polychaeta	
class	Pycnogonida	
class	Stenolaemata	
order	Actiniaria	
order	Amphipoda	
order	Anthoathecata	
order	Arcida	
order	Camarodonta	
order	Cheilostomatida	
order	Ctenostomatida	
order	Cyclostomatida	
order	Forcipulatida	
order	Gromiida	
order	Haplosclerida	
order	Isopoda	
order	Littorinimorpha	
order	Malacalcyonacea	
order	Mysida	
order	Neogastropoda	
order	Ophiurida	
order	Pantopoda	
order	Pectinida	
order	Phlebobranchia	
order	Phyllodocida	
order	Podocopida	
order	Poecilosclerida	
order	Sabellida	
order	Stolidobranchia	
order	Suberitida	
order	Tanaidacea	
order	Terebellida	
order	Trochida	
order	Valvatida	
family	Alcyonidiidae	
family	Alcyoniidae	
family	Amphilochoidae	
family	Arachnopusiidae	
family	Ascidiidae	
family	Asteriidae	
family	Beaniidae	
family	Bugulidae	
family	Calliopiidae	
family	Calliostomatidae	
family	Capulidae	
family	Celleporidae	
family	Chalinidae	
family	Clavulariidae	
family	Crisiidae	
family	Echinidae	
family	Eudendriidae	
family	Gromiidae	
family	Janiridae	
family	Laternulidae	
family	Lichenoporidae	
family	Microporidae	
family	Munnidae	
family	Mysidae	
family	Myxillidae	
family	Nototanaidae	
family	Odontasteridae	
family	Ophiopyrgidae	
family	Orbiniidae	
family	Pectinidae	
family	Philobryidae	
family	Philoporidae	
family	Polynoidae	
family	Prosiphonidae	
family	Rissoidae	
family	Romancheinidae	
family	Serpulidae	
family	Spirorbidae	
family	Styelidae	
family	Suberitidae	
family	Terebellidae	
family	Tubuliporidae	
family	Xestoleberididae	
genus	*Adamussium* Thiele, 1934	
genus	*Alcyonidium* Lamouroux, 1813	
genus	*Alcyonium* Linnaeus, 1758	
genus	*Antarcticaetos* Hayward & Thorpe, 1988	
genus	*Arachnopusia* Jullien, 1888	
genus	*Ascidia* Linnaeus, 1767	
genus	*Austrofilius* Hodgson, 1910	
genus	*Barrukia* Bergström, 1916	
genus	*Beania* Johnston, 1840	
genus	*Camptoplites* Harmer, 1923	
genus	*Clavularia* Blainville, 1830	
genus	*Cnemidocarpa* Huntsman, 1913	
genus	*Crisia* Lamouroux, 1812	
genus	*Cryocapulus* Schiaparelli, Bouchet, Fassio & Oliverio, 2020	
genus	*Diplasterias* Perrier, 1891	
genus	*Disporella* Gray, 1848	
genus	*Eucranta* Malmgren, 1865	
genus	*Eudendrium* Ehrenberg, 1834	
genus	*Exidmonea* David, Mongereau & Pouyet, 1972	
genus	*Favosthimosia* Hayward & Winston, 2011	
genus	*Filicrisia* d'Orbigny, 1853	
genus	*Gitanopsilis* Rauschert, 1994	
genus	*Gromia* Dujardin, 1835	
genus	*Haliclona* Grant, 1841	
genus	*Harmothoe* Kinberg, 1856	
genus	*Helicosiphon* Gravier, 1907	
genus	*Homaxinella* Topsent, 1916	
genus	*Klugeflustra* Moyano, 1972	
genus	*Lanicides* Hessle, 1917	
genus	*Laternula* Röding, 1798	
genus	*Leodamas* Kinberg, 1866	
genus	*Margarella* Thiele, 1893	
genus	*Micropora* Gray, 1848	
genus	*Munna* Krøyer, 1839	
genus	*Mysidetes* Holt & Tattersall, 1906	
genus	*Neobuccinum* E. A. Smith, 1879	
genus	*Nototanais* Richardson, 1906	
genus	*Odontaster* Verrill, 1880	
genus	*Ophioplinthus* Lyman, 1878	
genus	*Oradarea* Walker, 1903	
genus	*Philobrya* J. G. Cooper, 1867	
genus	*Powellisetia* Ponder, 1965	
genus	*Reteporella* Busk, 1884	
genus	*Serpula* Linnaeus, 1758	
genus	*Spirorbis* Daudin, 1800	
genus	*Stelodoryx* Topsent, 1904	
genus	*Sterechinus* Koehler, 1901	
genus	*Subonoba* Iredale, 1915	
genus	*Xestoleberis* Sars, 1866	
species	*Adamussiumcolbecki* (E. A. Smith, 1902)	
species	*Alcyoniumantarcticum* Wright & Studer, 1889	
species	Alcyoniumcf.antarticum Wright & Studer, 1889	
species	*Antarcticaetosbubeccata* (Rogick, 1955)	
species	*Arachnopusiadecipiens* Hayward & Thorpe, 1988	
species	Arachnopusiacf.decipiens Hayward & Thorpe, 1988	
species	*Austrofiliusfurcatus* Hodgson, 1910	
species	Barrukiacf.cristata (Willey, 1902)	
species	*Beaniaerecta* Waters, 1904	
species	*Camptoplitesbicornis* (Busk, 1884)	
species	*Camptoplitestricornis* (Waters, 1904)	
species	*Clavulariafrankliniana* Roule, 1902	
species	Clavulariacf.frankliniana Roule, 1902	
species	*Cnemidocarpaverrucosa* (Lesson, 1830)	
species	Cnemidocarpacf.verrucosa (Lesson, 1830)	
species	*Cryocapulussubcompressus* (Pelseneer, 1903)	
species	*Diplasteriasbrucei* (Koehler, 1907)	
species	*Eudendriumscotti* Puce, Cerrano & Bavestrello, 2002	
species	*Favosthimosiamilleporoides* (Calvet, 1909)	
species	*Gitanopsilisamissio* Rauschert, 1994*	
species	Gromiacf.melinus Rothe, Gooday, Cedhagen, Fahrni, Hughes, Page, Pearce & Pawlowski, 2009	
species	Harmothoecf.exanthema (Grube, 1856)	
species	Harmothoecf.spinosa Kinberg, 1856	
species	*Harmothoeantarctica* (McIntosh, 1885)	
species	*Harmothoecrosetensis* (McIntosh, 1885)	
species	Harmothoecf.crosetensis (McIntosh, 1885)	
species	*Harmothoefuligineum* (Baird, 1865)	
species	Harmothoecf.fuligineum (Baird, 1865)	
species	*Harmothoefullo* (Grube, 1878)	
species	*Homaxinellabalfourensis* (Ridley & Dendy, 1886)	
species	Xestoleberiscf.meridionalis Müller, 1908	
species	Klugeflustracf.vanhoeffeni (Kluge, 1914)	
species	*Lanicidesbilobata* (Grube, 1877)	
species	*Laternulaelliptica* (P. P. King, 1832)	
species	*Leodamasmarginatus* (Ehlers, 1897)	
species	*Margarellacrebrilirulata* (E. A. Smith, 1907)	
species	*Margarellarefulgens* (E. A. Smith, 1907)	
species	Microporacf.notialis Hayward & Ryland, 1993	
species	*Munnaantarctica* (Pfeffer, 1887)	
species	*Mysidetesilligi* Zimmer, 1914*	
species	*Neobuccinumeatoni* (E. A. Smith, 1875)	
species	*Nototanaisantarcticus* (Hodgson, 1902)	
species	*Nototanaisdimorphus* (Beddard, 1886)	
species	*Odontasterroseus* Janosik & Halanych, 2010	
species	*Odontastervalidus* Koehler, 1906	
species	*Ophioplinthusgelida* (Koehler, 1901)	
species	*Oradareaacuminata* Thurston, 1974	
species	*Philobryasublaevis* Pelseneer, 1903	
species	*Powellisetiadeserta* (E. A. Smith, 1907)	
species	*Stelodoryxcribrigera* (Ridley & Dendy, 1886)*	
species	*Sterechinusneumayeri* (Meissner, 1900)	
species	*Subonobagelida* (E. A. Smith, 1907)	

## Temporal coverage

**Data range:** 2015-12-01 – 2022-11-08.

## Collection data

### Collection name

MNA – Biological Collections

### Collection identifier

https://www.gbif.org/grscicoll/collection/a57a1dc1-706c-42db-bbad-1e68d9685439

### Parent collection identifier

Italian National Antarctic Museum (section of Genoa)

### Specimen preservation method

Specimens in 96% ethanol, slides with whole or dissected organisms (fixed in glycerol), part of organisms preserved in dry for SEM and frozen at -20°C.

## Usage licence

### Usage licence

Other

### IP rights notes

The dataset was published under the licence CC-BY 4.0.

## Data resources

### Data package title

Checklist of pioneer benthic taxa found on Autonomous Reef Monitoring Structures (ARMS) in Terra Nova Bay (Ross Sea, Antarctica)

### Resource link

https://doi.org/10.15468/4c7zf8

### Alternative identifiers

https://www.gbif.org/dataset/f024a3d3-7bb2-4985-be57-d890936769ff

### Number of data sets

1

### Data set 1.

#### Data set name

Checklist of pioneer benthic taxa found on Autonomous Reef Monitoring Structures (ARMS) in Terra Nova Bay (Ross Sea, Antarctica)

#### Data format

Darwin Core

#### Description

The dataset comprises a total of 277 distributional records, each one corresponding to a voucher specimen stored at the MNA, Section Genoa (Cometti et al. 2024). These records originate from ARMS, at varying time ranges of 1, 2, 3 and 5 years in Tethys Bay (Ross Sea, Antarctica). The occurrences presented in this dataset represent an important contribution as a baseline of the taxonomic composition of pioneer benthic communities in the Ross Sea and it will be useful to study their future dynamics.

**Data set 1.** 

Column label	Column description
occurrenceID	A global unique identifier for the occurrence.
institutionCode	The name (or acronym) in use by the institution having custody of the object(s) or information referred to in the record.
institutionID	An identifier for the institution having custody of the objects or information referred to in the record.
collectionCode	The acronym identifying the collection or dataset from which the record was derived.
collectionID	An identifier for the dataset from which the record was derived.
catalogNumber	An identifier of any form assigned by the source within a physical collection or digital dataset for the record which may not be unique, but should be fairly unique in combination with the institution and collection code.
basisOfRecord	The specific nature of the data record (Preserved Specimen).
type	The genre of the resource (PhysicalObject).
scientificName	The full scientific name, with authorship and date information, if known.
taxonRank	The taxonomic rank of the most specific name in the scientificName.
kingdom	The full scientific name of the kingdom in which the taxon is classified.
phylum	The full scientific name of the phylum in which the taxon is classified.
class	The full scientific name of the class in which the taxon is classified.
order	The full scientific name of the order in which the taxon is classified.
family	The full scientific name of the family in which the taxon is classified.
genus	The full scientific name of the genus in which the taxon is classified.
specificEpithet	The name of the first or species epithet of the scientificName.
scientificNameAuthorship	The authorship information for the scientificName formatted according to the conventions of the applicable.
identificationQualifier	A controlled value to express the determiner's doubts about the Identification (sp. cf).
scientificNameID	An identifier for the nomenclatural (not taxonomic) details of a scientific name.
individualCount	The number of individuals present at the time of the Occurrence.
lifeStage	The life stage of organisms. In detail, juveniles and eggs.
occurrenceRemarks	Antarctic Expeditions in which the organisms were sampled.
eventDate	The date-time or interval during which an Event occurred.
sampleSizeValue	A numerical value indicating the time of colonisation.
sampleSizeUnit	The unit of measurement of the time duration. This term must have a corresponding to sampleSizeValue.
eventID	A global unique identifier for the set of information associated with an Event.
decimalLatitude	The geographic latitude (in decimal degrees, using the spatial reference system given in geodeticDatum).
decimalLongitude	The geographic longitude (in decimal degrees, using the spatial reference system given in geodeticDatum).
geodeticDatum	The spatial reference system (WGS84) upon which the geographic coordinates given in decimalLatitude and decimalLongitude are based.
minimumDepthInMetres	Minimum sampling depth during event in metres.
maximumDepthInMetres	Maximum sampling depth during event in metres.
coordinatePrecision	A decimal representation of the precision of the coordinates given in the decimalLatitude and decimalLongitude.
samplingProtocol	Gear used to collect specimens and relative DOI of manuscript in which the sampling method is described.
dynamicProperties	Concatenation of information, specifically: Movement (sessile, vagile), PVC plate number (lowest = 1 to highest = 10) and plate orientation (T = top, B = bottom).
preparations	Description of the tissue or the voucher specimen and preservation method.
occurrenceStatus	Statement about the presence or absence of a specimen.
locality	The specific description of the place.
continent	Continent where the organisms were sampled.
countryCode	The standard code for the country where the organisms were sampled.
recordedBy	Surname and name of the personnel who collected the samples.
recordedByID	ORCID of the personnel who collected the samples.
identifiedBy	Surname and name of the personnel who analysed and recognised the single species.
identifiedByID	ORCID of the personnel who analysed and recognised the single species.
